# Correlation between CEUS LI-RADS categorization of HCC < 20 mm and clinic-pathological features

**DOI:** 10.1186/s13244-024-01688-7

**Published:** 2024-05-07

**Authors:** Daohui Yang, Xuejun Chen, Linjin Huang, Xi Wang, Lijuan Mao, Lewu Lin, Hong Han, Qing Lu

**Affiliations:** 1 Department of Ultrasound, Zhongshan Hospital (Xiamen), Fudan University, Xiamen, China; 2 Department of Ultrasound, Zhongshan Hospital, Institute of Ultrasound in Medicine and Engineering, Fudan University, Shanghai, China; 3 Shanghai Institute of Medical Imaging, Shanghai, China

**Keywords:** Hepatocellular carcinoma, Ultrasound, Contrast media, Diagnosis

## Abstract

**Objective:**

To retrospectively evaluate the diagnostic performance of contrast-enhanced ultrasound (CEUS) LI-RADS in liver nodules < 20 mm at high risk of hepatocellular carcinoma (HCC) and their correlation with clinic-pathological features.

**Methods:**

A total of 432 pathologically proved liver nodules < 20 mm were included from January 2019 to June 2022. Each nodule was categorized as LI-RADS grade (LR)-1 to LR-5 through LR-M according to CEUS LI-RADS. The sensitivity, specificity, positive predictive value (PPV), negative predictive value (NPV), and area under the curve (AUC) of CEUS LI-RADS were evaluated using pathological reference standard. Correlations between clinic-pathological features and CEUS LI-RADS categorization, together with major CEUS features, were further explored.

**Results:**

With LR-5 to diagnose HCC, the sensitivity, specificity, PPV, NPV, and AUC were 50.3%, 70.0%, 91.2%, 18.5%, and 0.601, respectively. The proportion of LR-5 in primary HCCs was significantly higher than that in recurrent ones (*p* = 0.014). HCC 10–19 mm showed significantly more frequent arterial phase hyper-enhancement (APHE) and late washout (*p* < 0.05) and less no-washout (*p* = 0.003) compared with those in HCC < 10 mm. Well-differentiated HCCs showed more frequent non-APHE and no-washout than moderate- and poor-differentiated HCCs (*p* < 0.05). Upgrading “APHE without washout” LR-4 nodules 10–19 mm with HCC history and “APHE with late mild washout” LR-4 nodules < 10 mm to LR-5 could improve the diagnostic performance of LR-5. The corresponding sensitivity, specificity, PPV, NPV, and AUC are 60.2%, 70.0%, 92.6%, 22.1%, and 0.651, respectively.

**Conclusions:**

CEUS LI-RADS is valuable in the diagnosis of HCC < 20 mm and performance can be improved with the combination of clinic-pathological features.

**Critical relevance statement:**

CEUS LI-RADS was valuable in the diagnosis of HCC < 20 mm and its diagnostic performance can be improved by combining clinic-pathological features. Further research is needed to define its value in this set of lesions.

**Key Points:**

- Contrast-enhanced ultrasound can detect small liver lesions where LI-RADS accuracy is uncertain.
- Many LI-RADS Grade-4 nodules were upgraded to Grade-5 by combining imaging with clinic-pathological factors.
- The reclassification of LI-RADS Grade-5 can improve sensitivity without decreasing positive predictive value.

**Graphical Abstract:**

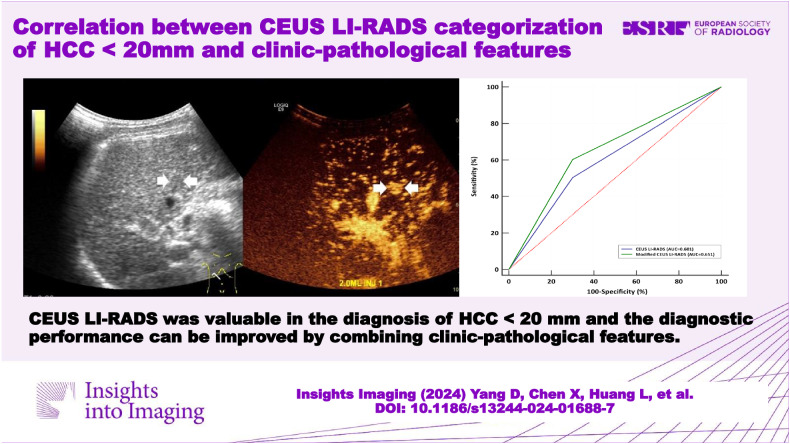

## Introduction

Hepatocellular carcinoma (HCC), which constitutes the most common primary liver malignancy, accounting for approximately 80%, is the sixth most diagnosed cancer and the fourth leading cause of cancer death worldwide [1, 2]. Imaging surveillance for high-risk HCC populations is recommended worldwide. Contrast-enhanced ultrasound (CEUS), a valuable technique for characterizing no more than two focal liver lesions usually during one examination, has been endorsed by German and Japanese HCC guidelines as a first-line diagnostic tool and by Asian, Italian, and updated European guidelines as a second-line tool after CT or MRI when they are contraindicated or their findings are inconclusive [3–6]. In 2017, the American College of Radiology developed the contrast-enhanced ultrasound Liver Imaging Reporting and Data System (CEUS LI-RADS) to overcome the observer dependency and subjectivity of CEUS and has become a useful tool during multidisciplinary discussions [7]. The performance of CEUS LI-RADS for the diagnosis of HCC has been analyzed in recent years with substantial study heterogeneity (proportion of HCC cases and the type of reference standard) with varied diagnostic value (sensitivity: 33–86%; specificity: 58–100%) [8, 9].

During routine screening for patients at high risk of HCC, together with the improvements in image modalities, liver nodules are prone to be detected at an earlier stage with relatively small size. However, the imaging of small HCC, especially those < 20 mm, is challenging because of the complexity of blood supply during multistage hepatic carcinogenesis, which leads to overlapping microvascular perfusion of regenerative and dysplastic nodules and HCC. Depending on the balance of remnant portal venules and new dysplastic arteries, small nodules may not demonstrate the classic HCC enhancement characteristics on CT or MRI, that is, arterial hyper-enhancement followed by washout in the portal-venous and delayed phase. The diagnostic performance of CEUS LI-RADS in characterizing small liver nodules (< 20 mm), to our best knowledge, has not been fully evaluated. The purpose of our study was to retrospectively evaluate the diagnostic performance of CEUS LI-RADS in liver nodules < 20 mm at high risk of HCC and to explore the correlation between clinic-pathological features and the CEUS LI-RADS categorization for further improving its diagnostic performance.

## Materials and methods

### Patient recruitment

**Fig. 1:**
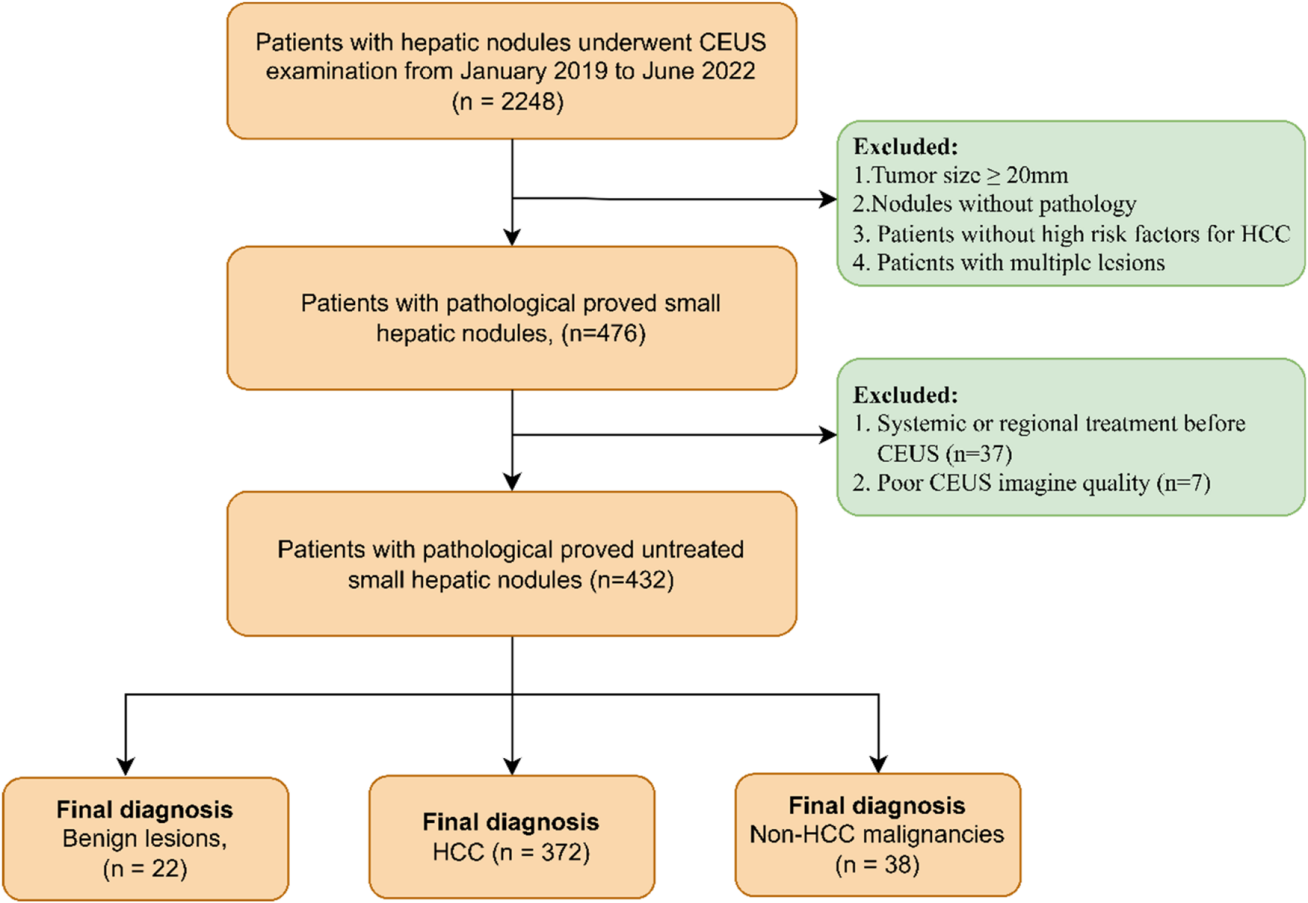
The flow chart of patient recruitment

The implementation of this retrospective observational study was approved by our institutional ethics committee (No. B2022-347R) and written informed consent was waived for its retrospective nature. Fig. 1 shows the flow chart of patient recruitment.

From January 2019 to June 2022, a total of 2248 consecutive patients with liver nodules underwent CEUS examination. Inclusion criteria were (1) pathological proved solitary nodule, either by percutaneous biopsy or by surgical resection; (2) with high-risk factor for HCC, including cirrhosis, chronic hepatitis B viral (HBV) or hepatitis C viral (HCV) infection, current or prior HCC; (3) visible distinct lesion on gray-scale ultrasound; (4) maximum diameter < 20 mm measured on gray-scale ultrasound; (5) available CEUS data within 2 weeks before surgery or biopsy; (6) no vascular invasion or thrombosis on imaging modalities, either CT/MRI or US. Exclusion criteria were as follows: (1) systemic or regional treatment before CEUS; (2) local relapse from previously treated lesions; (3) diffuse HCC; (4) patients with multiple lesions (*n* ≥ 2); (5) poor CEUS imaging quality (sever fatty liver or deep lesion location) or incomplete CEUS data (without either enhancement or washout features recorded on the hard disc).

### CEUS examination

Conventional ultrasound and CEUS examinations were performed by one of three board-certified radiologists with more than 10 years’ experience in abdominal US and CEUS (X.W., H.H., and Q.L., respectively) with GE Logiq E9 (GE Healthcare, Berlin, Germany), Phillip Epiq 7 (Philips Medical Solutions; Mountain View, CA, United States), or Hitachi-Aloka Preirus Arietta 70 (Tokyo, Japan). As a routine procedure of liver nodule examination, on gray-scale ultrasound, the location, number, and maximum diameter of nodules were recorded. The pulse inversion harmonic imaging was applied to CEUS examinations with a mechanical index < 0.1. A bolus injection of 1.2–2.4 mL sulfur hexafluoride-filled contrast agent (SonoVue; Bracco, Milan, Italy) was injected via an antecubital 20-gauge catheter followed by a 5-mL flush of 0.9% sodium chloride solution. A timer was started immediately at the end of the contrast agent injection, and each CEUS examination lasted at least 2 min, with recording arterial phase (10–30 s), portal-venous phase (30–120 s), and delayed phase (> 120 s) in digital video format for further evaluation.

### CEUS imaging interpretation

The CEUS images were assessed by two experienced radiologists with 5 years (D.Y.) and 8 years’ experience (X.C.) in liver CEUS interpretation in consensus, they were both blinded to the final pathology and clinical data. According to the American College of Radiology (ACR) CEUS LI-RADS v2017, the categorization was based on the arterial enhancement, onset time of washout, and washout degree at 120 s after contrast agent injection. In the arterial phase, the enhancement pattern was classified as follows: homogeneous or heterogeneous hyper-enhancement, rim-like hyper-enhancement, iso-enhancement, and hypo-enhancement. Both homogeneous and heterogeneous hyper-enhancement were referred to as arterial phase hyper-enhancement (APHE), and rim-like hyper-enhancement was referred to rim-APHE. As for washout, it was applied only in those with hyper-enhancement or iso-enhancement during the arterial phase. The degree of washout was categorized into three types: no washout, mild washout (a reduction in enhancement degree but continues to show some microbubbles within the nodule), and marked washout, compared with the surrounding liver parenchyma. According to the onset time of washout, early and late washout were defined with 60 s as the cut-off value. The above-mentioned CEUS features, together with lesion size, were used to give each lesion a CEUS LI-RADS categorization [10]. If no consensus was reached, the final categorization was achieved by another expert ultrasound physician with 15 years of experience in CEUS.

### Reference standard

The reference standard for the final diagnosis was histopathological findings, either by surgical resection (424/432) or by US-guided percutaneous biopsy (8/432). Surgical or biopsy specimens were assessed for hepatic background using the Scheuer fibrosis stage and for HCC grades using Edmondson-Steiner grades, which were routinely recorded on the pathological reports.

### Statistical analysis

Statistical analysis was performed with IBM SPSS Statistics version 22.0 (Armonk, NY, USA; IBM Corp) and MedCalc version 19.0.4 (MedCalc Software, Ltd, Ostend, Belgium). Qualitative data were presented as numbers and percentages, and quantitative data were presented as mean ± standard deviation. The chi-square test and Fisher’s exact test were used for the comparisons of categorical data. Student’s t-test for unpaired samples was used for comparison of mean values. For the performance of CEUS LR-5 and LR-M in the diagnosis of HCC and non-HCC malignancies, the sensitivity, specificity, positive predictive values (PPV), negative predictive values (NPV), and area under the curve (AUC) were calculated, respectively. Since CEUS was an imaging modality associated with certain observer experience and subjectivity, the Kappa value was calculated to measure the inter-observer agreement of CEUS LI-RADS categorization. The strength of agreement was interpreted as follows: 0–0.20, poor agreement; 0.21–0.40, fair agreement; 0.41–0.60, moderate agreement; 0.61–0.80, substantial agreement; and 0.81–1.00, almost perfect agreement. Differences were considered statistically significant for *p* < 0.05. To better understand the clinic-pathological features that might influence CEUS LI-RADS diagnostic performance on HCCs, the correlations between HCC grade, fibrotic stage of liver parenchyma, CEUS LI-RADS categorization, and major CEUS features were explored, respectively.

## Results

### Subject demographic characteristics

In total, 432 small solitary liver nodules < 20 mm in 432 patients were included in this retrospective study with a mean size 14.7 ± 3.5 mm (range 5–19 mm). Table 1 lists the demographic features of patients and liver nodules.

**Table 1:** Subject demographic characteristics

Variable	Value (%)
No. of men/women	350 (81.0%)/82 (19.0%)
Median age (years)*	58 (57.4 ± 9.7)
Size	
< 10 mm	50 (11.6%)
10–20 mm	382 (88.4%)
Recurrence	
Yes	153 (35.4%)
No	279 (64.6%)
Etiology	
HBV	431 (99.8%)
HCV	10 (2.3%)
Parenchymal background	
Fibrosis	401 (92.8%)
Cirrhosis	264 (61.1%)
Pathological diagnosis	
HCC	372 (86.1%)
ICC	8 (1.9%)
CHC	20 (4.6%)
MLT	6 (1.4%)
Other malignancies	4 (0.9%)
DN	3 (0.7%)
RN	1 (0.2%)
FNH	5 (1.2%)
Other benign nodules	13 (3.0%)
Differentiation of HCC	
Well-differentiated	6 (1.6%)
Moderate-differentiated	290 (78.0%)
Poor-differentiated	76 (20.4%)

Except where indicated, the data are the number of nodules, and the number in brackets is the percentage, *data are mean.

*ICC* intrahepatic cholangiocarcinoma, *MTL* metastatic liver tumors, *CHC* combined hepatocellular cholangiocarcinoma, *DN* dysplastic nodule, *RN* regenerative nodule, *FNH* focal nodular hyperplasia

### Inter-observer agreement in CEUS LI-RADS categorization

Inter-observer agreement according to Cohen’s Kappa was excellent on CEUS LI-RADS with the Kappa value of 0.830 (confidence interval [CI]: 0.788, 0.872). The results are summarized in Table 2.

**Table 2:** Inter-observer agreement in CEUS LI-RADS categorization

Reader 1	Reader 2					Total
	LR-2	LR-3	LR-4	LR-5	LR-M	
**LR-2**	5	1	0	0	0	6
**LR-3**	0	30	5	3	0	38
**LR-4**	0	3	65	2	4	74
**LR-5**	0	1	3	191	39	234
**LR-M**	0	0	0	1	79	80
**Total**	5	35	73	197	122	432

### CEUS LI-RADS categories and diagnostic performance

The distribution of the CEUS LI-RADS categorizations and pathological results are summarized in Table 3. The distribution of HCC was 0.0%, 1.1%, 8.1%, 16.4%, 50.2%, and 24.2% in CEUS LR-1, 2, 3, 4, 5, and M, respectively.

**Table 3:** The distribution of CEUS LI-RADS categorization and pathological diagnosis

Pathological diagnosis	LR-2	LR-3	LR-4	LR-5	LR-M	Total
HCC	4	30	61	187	90	372
ICC	0	1	1	3	3	8
MLT	0	0	1	0	5	6
CHC	0	0	3	6	11	20
DN	0	1	1	1	0	3
RN	0	1	0	0	0	1
FNH	0	1	4	0	0	5
Other malignant nodules	0	0	0	1	3	4
Other benign nodules	1	1	1	7	3	13
Total	5	35	72	205	115	432

CEUS LI-RADS, contrast-enhanced ultrasound Liver Imaging Reporting and Data System

**Fig. 2:**
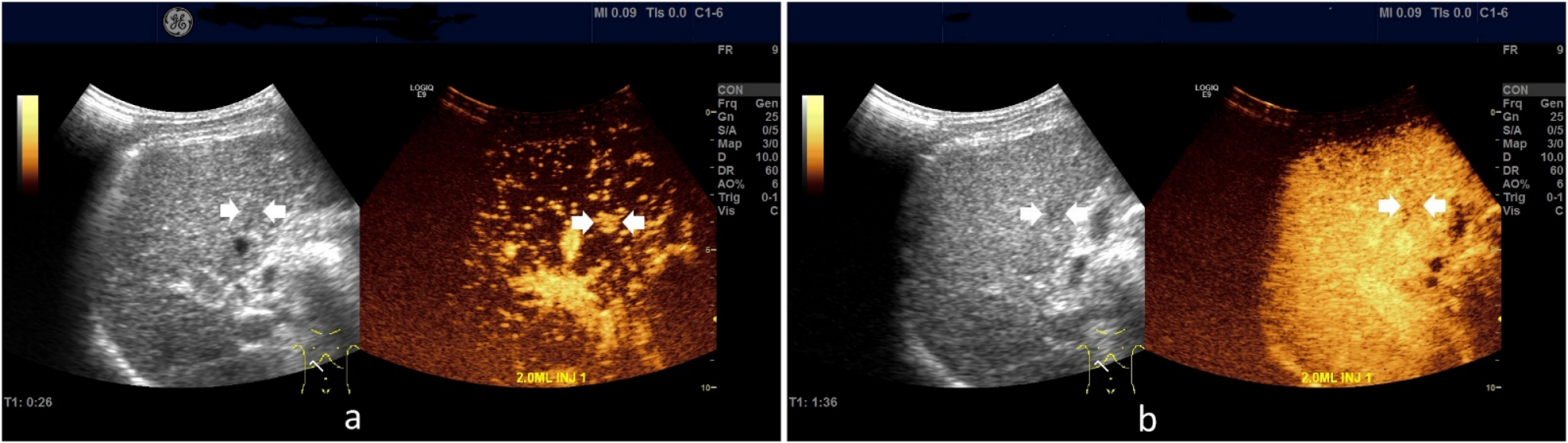
Images showed a nodule classified as LR-5 in a 62-year-old male with chronic hepatitis B virus-related cirrhosis and pathologically confirmed HCC. A 13 mm nodule located in the right lobe of the liver showed homogeneous hyperenhancement (arrow) in the arterial phase on contrast-enhanced ultrasound (**a**). A mild washout (arrow) was observed in the portal-venous phase (> 60 seconds; timer, 01:36) (**b**). The lesion was classified as LR-5 according to the CEUS LI-RADS.

LR-5 category included 187 (91.2%) HCCs (Fig. 2), 10 non-HCC malignancies (3 intrahepatic cholangiocarcinoma, 6 combined hepatocellular cholangiocarcinoma, 1 primary hepatic lymphoma) (4.9%), and 8 benign nodules (1 dysplastic nodule, 3 epithelioid angiomyolipomas, 2 focal inflammatory lesions, 1 hepatocellular adenoma and 1 intrahepatic bile duct adenoma) (3.9%). With LR-5 to diagnose HCC, the sensitivity, specificity, PPV, NPV, and AUC were 50.3% (95%CI: 45.1%, 55.5%), 70.0% (95%CI: 56.8%, 81.2%), 91.2% (95%CI: 87.4%, 93.9%), 18.5% (95%CI: 15.7%, 21.6%), and 0.601 (95%CI: 0.553, 0.648), respectively.

LR-M included 22 (19.1%) non-HCC malignancies, 90 (78.3%) HCCs, and 3 (2.6%) benign nodules (focal inflammatory lesions). With LR-M for the diagnosis of non-HCC malignancy, the sensitivity, specificity, PPV, NPV, and AUC were 57.9% (95%CI: 40.8%, 73.7%), 76.4% (95%CI: 71.9%, 80.5%), 19.1% (95%CI: 14.6%, 24.6%), 95.0% (95%CI: 92.8%, 96.5%), and 0.671 (95%CI: 0.625, 0.716), respectively.

### Correlations between HCC CEUS LI-RADS categorization and clinic-pathological features

The correlations between HCC CEUS LI-RADS categorization and clinic-pathological features are summarized in Table 4.

**Table 4:** Correlations between HCC CEUS LI-RADS categorization with clinic-pathological features

Category	HCC				
	LR-2	LR-3	LR-4	LR-5	LR-M
Size					
< 10 mm (*n* = 40)	4	13	16	0	7
10–19 mm (*n* = 332)	0	17	45	187	83
Recurrence					
Yes (*n* = 136)	2	15	30	57	32
No (*n* = 236)	2	15	31	130	58
Cirrhosis					
Presence (*n* = 249)	1	21	42	125	60
Absence (*n* = 123)	3	9	19	62	30
Differentiation					
Well (*n* = 6)	0	4	1	1	0
Moderate (*n* = 290)	3	22	47	149	69
Poor (*n* = 76)	1	4	13	37	21
Serum AFP level					
≥ 20 ng/mL (*n* = 145)	2	9	22	74	38
< 20 ng/mL (*n* = 227)	2	21	39	113	52
Total	4	30	61	187	90

The proportion of LR-5 categorization in primary HCCs was significantly higher than that in recurrent ones (55.1% vs. 41.9%, *p* = 0.014), and the proportion of LR-4 categorization was significantly higher in recurrent HCCs than that in primary ones (22.1% vs. 13.1%, *p* = 0.029). LR-M categorization in primary and recurrent HCCs made no significant difference (24.6% vs. 23.5%, *p* = 0.820).

The distribution of HCC in LR-5 and LR-M was not influenced by the hepatic background, HCC grade, and serum alpha-fetoprotein (AFP) level (all *p* > 0.05). According to CEUS LI-RADS, tumor size is a subsequent influence factor of categorization. Since the threshold of nodule size for LR-5 was 10 mm, no nodules < 10 mm could be categorized into LR-5. The distribution of HCC < 10 mm in CEUS LR-1, 2, 3, 4, and M was 0.0%, 10.0%, 32.5%, 40.0%, 0.0%, and 17.5%, respectively; and the distribution of HCC 10–19 mm was 0.0%, 0.0%, 5.1%, 13.6%, 56.3%, and 25.0%, respectively.

### Correlations between HCC major CEUS LI-RADS features and clinic-pathological features

The correlations between HCC major CEUS LI-RADS features and clinic-pathological features are illustrated in Table 5. HCC 10–19 mm showed a significantly higher frequency of APHE than HCC < 10 mm (92.5% vs. 82.5%, *p* = 0.034), HCC < 10 mm showed more frequent no-washout and less frequent late washout (37.5% vs. 17.8%, *p* = 0.003; 45.0% vs. 55.4%, *p* = 0.036) compared with HCC 10–19 mm. With respect to the influence of HCC grade on CEUS features, the prevalence of non-APHE and no-washout were substantially greater in well-differentiated HCCs than those in moderate- and poor-differentiated HCCs (50.0% vs. 7.9%, *p* = 0.010; 66.7% vs. 19.1%, *p* = 0.016). The other clinical-pathological features, including hepatic background, AFP, and recurrence or primary HCC did not correlate with the CEUS LI-RADS major features of HCCs.

**Table 5:** Correlation between major CEUS LI-RADS features and clinic-pathological features of HCC

	Arterial phase		Portal and delayed phase				
	Non-APHE	APHE	Non-WO		Early WO		Late WO
Size							
< 10 mm (*n* = 40)	7 (17.5%)	33 (82.5%)	15 (37.5%)		7 (17.5%)		18 (45.0%)
10–19 mm (*n* = 332)	25 (7.5%)	307 (92.5%)	59 (17.8%)		89 (26.8%)		184 (55.4%)
*p* value	0.034		0.012				
Recurrence							
Yes (*n* = 135)	12 (8.9%)	123 (91.1%)	35 (25.9%)		32 (23.7%)		68 (50.4%)
No (*n* = 237)	20 (8.4%)	217 (91.6%)	40 (16.9%)		64 (27.0%)		133 (56.1%)
* p* value	0.882		0.154				
Differentiation							
Well (*n* = 6)	3 (50.0%)	3 (50.0%)	4 (66.7%)		0 (0.0%)		2 (33.3%)
Moderate (*n* = 290)	20 (6.9%)	270 (93.1%)	55 (19.0%)		75 (25.8%)		160 (55.2%)
Poor (*n* = 76)	9 (11.8%)	67 (88.2%)	15 (19.8%)		21 (27.6%)		40 (52.6%)
* p* value	0.004		0.126				
Cirrhosis							
Yes (*n* = 249)	19 (7.6%)	230 (92.4%)	49 (19.7%)		65 (26.1%)		135 (54.2%)
No (*n* = 123)	13 (10.6%)	110 (89.4%)	25 (20.3%)		31 (25.2%)		67 (54.5%)
* p* value	0.342		0.978				
Serum AFP level							
≥ 20 ng/mL (*n* = 148)	11 (7.4%)	137 (92.6%)	22 (14.9%)		41 (27.7%)		85 (57.4%)
< 20 ng/mL (*n* = 224)	21 (9.4%)	203 (90.6%)	52 (23.2%)		55 (24.6%)		117 (52.2%)
* p* value	0.513		0.142				

*APHE* arterial phase hyper-enhancement, *WO* washout

**Fig. 3:**
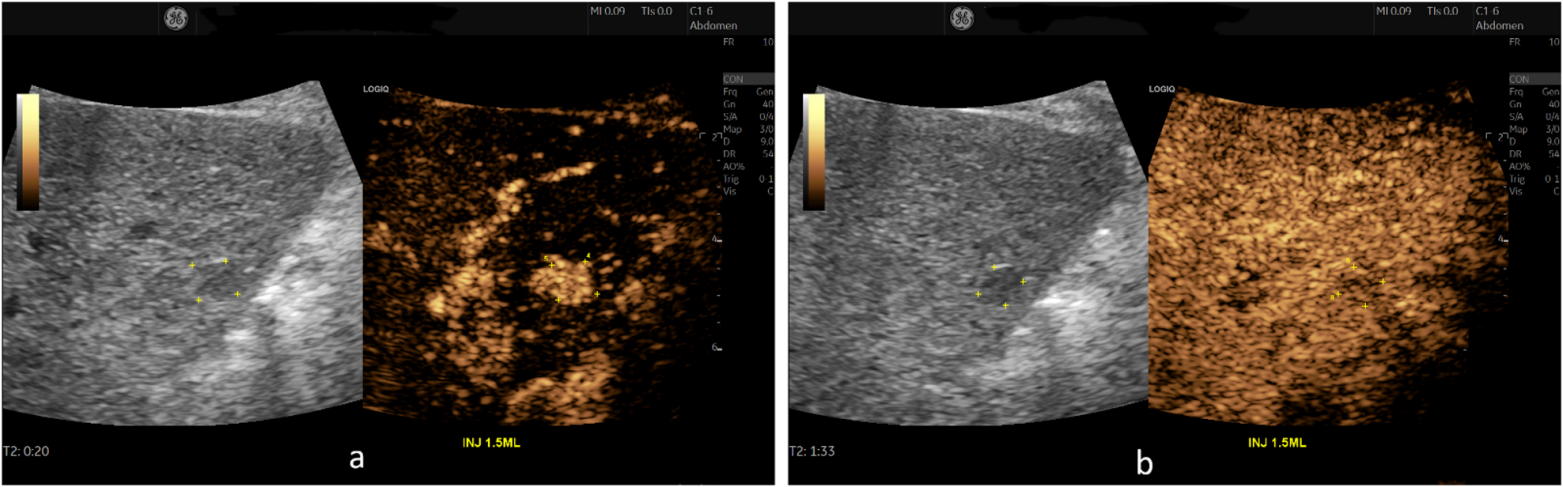
Images in a 61-year-old male with chronic hepatitis B virus-related cirrhosis and HCC history. A 9 mm nodule in the right lobe of the liver showed homogeneous hyperenhancement in the arterial phase on CEUS (**a**). A mild washout was observed in the portal-venous phase (> 60 seconds; timer, 01:33) (**b**). The lesion was classified as LR-4 according to the CEUS LI-RADS. While this lesion was recategorized to LR-5 according to the features of “APHE and late mild washout” and it was finally confirmed as hepatocellular carcinoma by histopathology

**Fig. 4:**
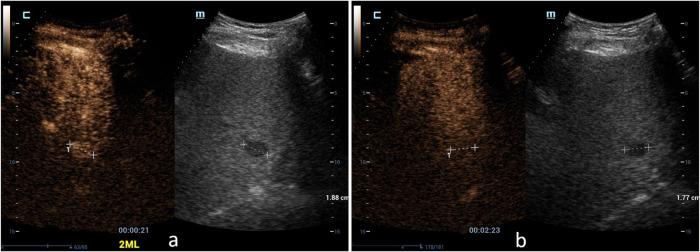
A nodule in a 42-year-old male with an HCC history was classified as LR-4 according to the CEUS LI-RADS. A 19 mm nodule located in the right lobe of the liver was homogeneous hyperenhanced in the arterial phase on CEUS (**a**). Washout was not observed during the portal-venous phase to the late phase (> 120 seconds) (**b**). We upgraded this lesion that showed “APHE with no washout” with HCC history from LR-4 to LR-5, and this lesion was finally confirmed as hepatocellular carcinoma by histopathology.

**Fig. 5:**
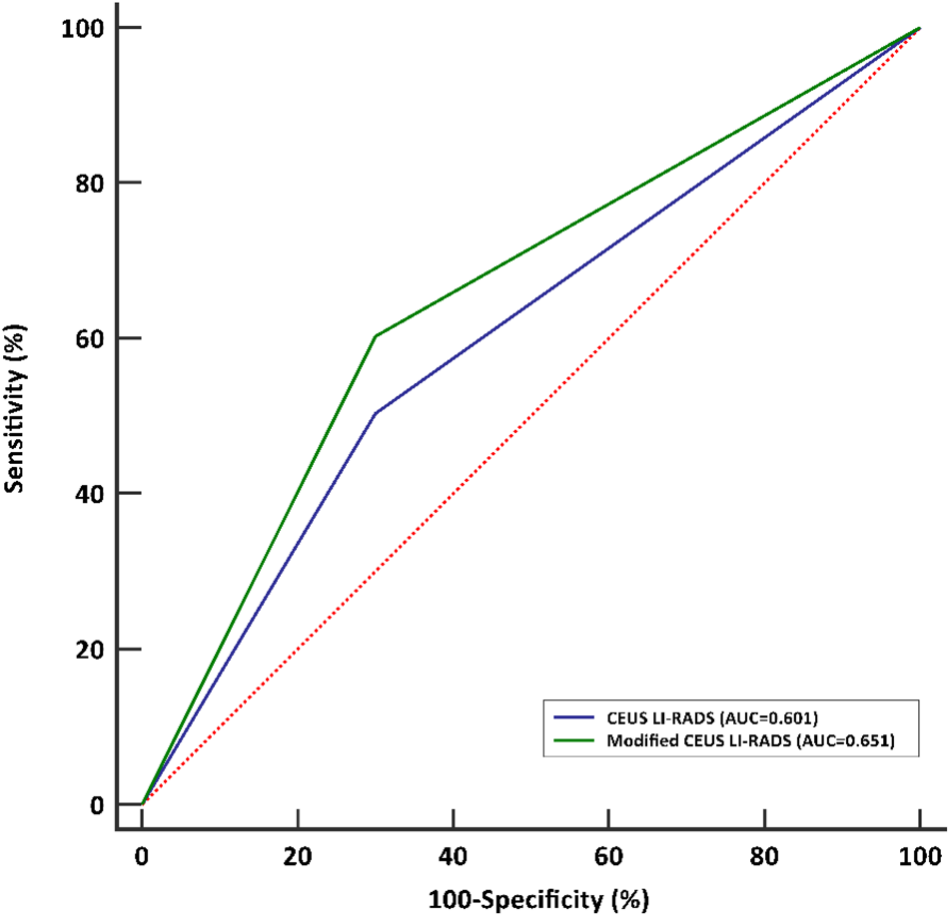
The compassion of area under the receiver operating characteristic (ROC) curve of CEUS LI-RADS (blue line) and modified CEUS LI-RADS (green line) for diagnosing HCC < 20 mm. DeLong’s tests show that the *p* value between the ROC curve of CEUS LI-RADS and modified CEUS LI-RADS was less than 0.001.

With upgrading LR-4 nodules < 10 mm with “APHE and late mild washout” to LR-5 (*n* = 19) (Fig. 3), 16/19 HCC might be correctly categorized into LR-5. CEUS LI-RADS categorization with upgrading LR-4 nodules 10–19 mm with HCC history and “APHE with no washout” to LR-5 (*n* = 22) (Fig. 4), 22/22 HCCs might be correctly categorized into LR-5. Then the diagnostic performance of LR-5 in the diagnosis of HCC would be improved, with sensitivity, specificity, PPV, NPV, and AUC being 60.2% (95%CI: 55.3%, 65.5%), 70.0% (95%CI: 56.8%, 81.2%), 92.6% (95%CI: 89.4%, 94.9), 22.1% (95%CI: 18.8%, 26.0%), and 0.651 (95%CI: 0.596, 0.698), respectively. The AUC (0.651) of LR-5 in modified CEUS LI-RADS was significantly higher than that (0.601) in CEUS LI-RADS (*p* < 0.001) (Fig. 5).

## Discussion

With the decrease in liver nodule diameter, the diagnostic sensitivity of imaging techniques would correspondingly reduce from 79–97% in HCC ≥ 20 mm [11, 12] to 52–67% in HCC < 20 mm [13, 14] since small liver nodules usually possess atypical imaging features. Therefore, it is urgent to improve the diagnostic performance of imaging modalities in small liver nodules. Although CEUS with real-time dynamic observation has the ability to highlight the tumor’s microvascular perfusion, it cannot be considered the diagnostic technique in all cases. Regenerative nodule and classic HCCs have distinctive enhancement patterns, while dysplastic nodule and well-differentiated HCC are highly variable with many combinations of arterial and portal venous phase appearances, which is in concordance with our results.

In present study, the sensitivity and specificity of LR-5 for HCC diagnosis were 50.3% and 70.0%, respectively, which was significantly lower than that in Peng’s meta-analysis for nodules < 20 mm (sensitivity 0.70 [95%CI 0.64–0.76], specificity 0.91 [95%CI 0.87–0.95]) [15]. We hypothesized that the relatively lower sensitivity and specificity in this study might be caused by two factors: First, the difference of reference standard (pathology in our study vs. composite clinical reference standard [CCRS] in Peng’s study). Since studies with pathology as a reference standard might contain more atypical HCCs, the diagnostic performance of CEUS LI-RADS has been reported to be significantly lower than that employing CCRS [8]. Second, the difference in HCC grades. Moderate- and poorly differentiated HCC were more likely to be categorized into LR-5 or LR-M than well-differentiated HCC [16–19]. However, our study showed no correlation between CEUS LI-RADS categorization and HCC grade since only 6 well-differentiated HCCs were included. This should be further explored with more well-differentiated HCCs included. Furthermore, our present results showed that LR-5 category in primary HCC was more common than that in recurrent HCC, while LR-4 HCCs were more common in recurrent ones. Therefore, the clinic-pathological features might influence the CEUS LI-RADS categorization of HCCs < 20 mm.

In the study by Terzi et al [20], CEUS LI-RADS LR-3/4 HCCs were reported to have no better clinical outcome than typical LR-5 HCCs, which emphasized the importance of minimizing the “downgrading” of HCC categorization to avoid leaving aggressive HCC without timely treatment [6]. Since the CEUS LI-RADS categorization is based on the combination of nodule size and major CEUS features, in this study, we further explored the correlations between CEUS features and clinic-pathological features. According to the CEUS LI-RADS algorithm, the threshold of nodule size for LR-5 was 10 mm, and no nodules < 10 mm could be categorized into LR-5, even with typical “APHE and late mild washout”. To our best knowledge, rare literature has ever included nodules < 10 mm for the evaluation of CEUS LI-RADS diagnostic performance. Here, 40 HCCs < 10 mm were included, and 72.5% of them were categorized into LR-3 or LR-4 according to the presence of APHE, together with washout onset time and degree. Since HCC < 10 mm commonly showed no-washout compared with HCC 10–19 mm, after upgrading LR-4 nodules < 10 mm with typical “APHE with late mild washout” to LR-5, 16/19 LR-4 HCCs were correctly categorized into LR-5. Furthermore, we found that no-washout was more common in recurrent HCCs than those without prior HCC history. Thus, we upgraded LR-4 nodules 10–19 mm demonstrating “APHE and no-washout” with prior HCC history to LR-5, and 22/22 HCCs were correctly categorized. Therefore, with the combination of clinic-pathological features to upgrade these two types of LR-4 nodules, the modified CEUS LI-RADS lead to a significantly improved sensitivity (from 50.3% to 60.2%) without a corresponding reduction in specificity (70.0% for each) or PPV (91.2%, and 92.6%, respectively). This would be helpful for physicians to choose the optimal treatment strategies as early as possible to improve prognosis.

LR-M was designed to preserve the specificity for the diagnosis of HCC while not losing sensitivity for the diagnosis of malignancy. In this study, 115 nodules were categorized in CEUS LR-M, which had a high proportion of HCC (78.3%). With LR-M to diagnose non-HCC malignancies, the sensitivity and specificity were 57.9 and 75.8%, respectively. Other studies showed that 25–63% of LR-M lesions were HCC with various nodule size range and percentages of ICC and CHC in each study [21–23]. In the present study, the imaging features of LR-M HCC were rim-APHE (6.7%), and early washout (< 60 s) (98.9%). From this point of view, early washout may be the main reason for the LR-M classification. Chen et al also reported that early washout was more common in CEUS for HCC classified into LR-M compared with the marked one [24]. We made an effort to explore the correlation between early washout (< 60 s) and clinic-pathological features. Unfortunately, no significant correlation was detected, though poor differentiation of HCC has been previously reported to be related to early washout. This discrepancy may be related to the relatively small sample size of non-HCC malignancy in our study (*n* = 38). Nowadays, efforts have been extended by modifying the LR-M criteria to improve the differentiation between HCCs and non-HCC malignancies, such as resetting the threshold for late washout (> 45 s) [25], and using additional image features [26]. However, the LR-M criteria still needs further modification to improve the diagnostic performance of CEUS LI-RADS.

Our study also had some limitations. First, due to the topic focusing on small liver nodules (< 20 mm) and its retrospective nature with single-center design, our result is placed at a lower level in the hierarchy of evidence-based medicine, and the relatively small sample size of nodules that upgraded from LR-4 to LR-5 can introduce a bias and limited the generalization of our results. Second, using pathology as a reference standard exclusively might introduce verification bias because lesions receiving pathological assessment are more inclined to be malignant and be assigned to higher CEUS LI-RADS categories. However, choosing a pathological reference standard seems the best way to enable an objective assessment of diagnostic accuracy compared with CCRS. Third, chronic HBV infection accounted for the major etiology of HCC in the present study. Thus, our results may not be reproducible in patients with other etiologic causes (non-alcoholic steatohepatitis) or the Western population (HCV infection). Further prospective multicenter studies should be investigated in the future. Fourth, there were seven patients with poor CEUS imaging quality were excluded from the present study, which would introduce a bias and a strong limitation to clinical practice.

In conclusion, the present data supported the value of CEUS LR-5 for HCC < 20 mm. With the combination of clinic-pathological features, the diagnostic performance of CEUS LI-RADS in HCC < 20 mm can be improved.

## Data Availability

The datasets used or analyzed during the current study are available from the corresponding author on reasonable request.

## Abbreviations

AUC: Area under the curve
APHE: Arterial phase hyper-enhancement
CCRS: Composite clinical reference standard
CEUS: Contrast-enhanced ultrasound
HBV: Hepatitis B viral infection
HCV: Hepatitis C viral infection
HCC: Hepatocellular carcinoma
LR: LI-RADS category
LI-RADS: Liver Imaging Reporting and Data System
NPV: Negative predictive value
PPV: Positive predictive value
